# Legume Alternative Oxidase Isoforms Show Differential Sensitivity to Pyruvate Activation

**DOI:** 10.3389/fpls.2021.813691

**Published:** 2022-01-17

**Authors:** Crystal Sweetman, Jennifer Selinski, Troy K. Miller, James Whelan, David A. Day

**Affiliations:** ^1^College of Science and Engineering, Flinders University, Bedford Park, SA, Australia; ^2^Department of Plant Cell Biology, Botanical Institute, Christian-Albrecht University of Kiel, Kiel, Germany; ^3^Department of Animal, Plant, and Soil Science, School of Soil Science, La Trobe University, Bundoora, VIC, Australia

**Keywords:** alternative oxidase, isoform, recombinant, kinetics, pyruvate, activation, soybean, legume

## Abstract

Alternative oxidase (AOX) is an important component of the plant respiratory pathway, enabling a route for electrons that bypasses the energy-conserving, ROS-producing complexes of the mitochondrial electron transport chain. Plants contain numerous isoforms of AOX, classified as either AOX1 or AOX2. AOX1 isoforms have received the most attention due to their importance in stress responses across a wide range of species. However, the propensity for at least one isoform of AOX2 to accumulate to very high levels in photosynthetic tissues of all legumes studied to date, suggests that this isoform has specialized roles, but we know little of its properties. Previous studies with sub-mitochondrial particles of soybean cotyledons and roots indicated that differential expression of GmAOX1, GmAOX2A, and GmAOX2D across tissues might confer different activation kinetics with pyruvate. We have investigated this using recombinantly expressed isoforms of soybean AOX in a previously described bacterial system (Selinski et al., 2016, *Physiologia Plantarum* 157, 264-279). Pyruvate activation kinetics were similar between the two GmAOX2 isoforms but differed substantially from those of GmAOX1, suggesting that selective expression of AOX1 and 2 could determine the level of AOX activity. However, this alone cannot completely explain the differences seen in sub-mitochondrial particles isolated from different legume tissues and possible reasons for this are discussed.

## Introduction

It is well documented that the plant mitochondrial electron transport chain (mETC) is branched, with phosphorylating and non-phosphorylating pathways coexisting (see Siedow and Day, 2000 for an overview). In addition to the classical mETC linked to proton translocation and ATP synthesis, plant mitochondria possess an alternative pathway that is non-proton translocating and consists of external and internal NAD(P)H dehydrogenases (NDBs and NDAs, respectively) and the so-called alternative oxidase (AOX), which accepts electrons from ubiquinol and provides an alternative route for electrons to reduce oxygen. It has been proposed based on co-expression analysis that NDB2, the major external NADH dehydrogenase, and AOX could potentially form a complete respiratory chain (Clifton et al., 2005). A functional link has been recently confirmed between the two proteins, which together help the plant to cope with the oxidative stress associated with photo inhibitory conditions (Sweetman et al., 2019b). An important role for AOX in minimizing oxidative stress under many different conditions has been shown by numerous studies (Maxwell et al., 1999; Pastore et al., 2001; Umbach et al., 2005; Smith et al., 2009; Cvetkovska and Vanlerberghe, 2013; Li et al., 2013; Mhadhbi et al., 2013; Vishwakarma et al., 2015; Dahal and Vanlerberghe, 2017; Demircan et al., 2020; Jayawardhane et al., 2020). However, operation of the alternative pathway has the potential to dramatically decrease the yield of ATP during respiration (Ribas-Carbo et al., 2005; Soole and Sweetman, 2021) and it is important that it is regulated carefully.

The *in vivo* activity, or engagement, of alternative oxidase (AOX) in plants reflects many levels of regulation, including transcriptional and post-transcriptional control. AOX capacity may be limited by protein abundance (e.g., Elthon and McIntosh, 1987; Rhoads and McIntosh, 1992; Vanlerberghe and McIntosh, 1994; McCabe et al., 1998), but increasing protein abundance does not always result in increased capacity nor activity (Obenland et al., 1990; Guy and Vanlerberghe, 2005; Florez-Sarasa et al., 2011). Protein abundance may or may not be related to gene expression (Rhoads and McIntosh, 1992; Vanlerberghe and McIntosh, 1994, 1996; Cruz-Hernandez and Gomez-Lim, 1995; Aubert et al., 1997; McCabe et al., 1998), with discrepancies potentially due to the half-life of AOX proteins, some of which can remain long after their transcripts subside. Activity is dependent on dimerization of the 30–40 kDa monomers, which must be in the reduced (i.e., non-covalently bound) form (Umbach and Siedow, 1993), with the reduction state of AOX *in vivo* probably regulated by thioredoxin (but see Florez-Sarasa et al., 2019; Moller et al., 2020). Another layer of regulation is the provision of substrate to the mitochondria, since the reduction state of ubiquinol (Q) to a particular threshold (i.e., high QH_2_:Q), at least partly determines AOX activity (but see Millenaar and Lambers, 2003), depending on the activation status of the protein (Hoefnagel et al., 1995), which in turn is dependent on various metabolites. In particular, activity of AOX in the reduced dimer state is regulated by certain organic acids, most notably pyruvate and other 2-oxo acids (Millar et al., 1993, 1996; Selinski et al., 2017), which interact with key cysteine residues of AOX within the mitochondrial matrix (Umbach and Siedow, 1996; Rhoads et al., 1998; Vanlerberghe et al., 1998; Djajanegara et al., 1999), forming a thiohemiacetal group (Umbach and Siedow, 1996). The presence of such activators decreases the threshold of QH_2_:Q required for AOX engagement (Umbach et al., 1994; Hoefnagel et al., 1995). The *in vivo* relevance of these various layers of regulation have been debated in the literature (e.g., Day and Wiskich, 1995; Del-Saz et al., 2018; Schwarzlander and Fuchs, 2019).

Plant AOX proteins are classified into two subfamilies, AOX1 and AOX2 (Considine et al., 2002) and four phylogenetic clades: AOX1a-c/1e, AOX1d, AOX2a-c, and AOX2d (Costa et al., 2014). While most angiosperms possess an expanded AOX1 subfamily alongside a single AOX2, legumes generally exhibit a single AOX1 and an expanded AOX2 subfamily (Costa et al., 2014; Sweetman et al., 2019a). Across all plants studied to date, there is at least one stress-inducible gene, typically an *Aox1a, b* and/or *Aox1d* (Considine et al., 2002; Clifton et al., 2005; Polidoros et al., 2005; Feng et al., 2013; Wanniarachchi et al., 2018), although *Aox2d* in chickpea and other legumes is also responsive to cold, drought and salinity stresses (Costa et al., 2014; Sweetman et al., 2019a,^2020^). The genome of soybean (*Glycine max*) contains three AOX genes *GmAox1*, *GmAox2a*, and *GmAox2d* and these are preferentially expressed in different tissues. The most abundant AOX protein of soybean photosynthetic tissues, GmAOX2a, is absent in roots and this pattern of expression is conserved for orthologs in other legumes (Kearns et al., 1992; Finnegan et al., 1997; Sweetman et al., 2019a). Importantly, in contrast to non-legumes in which AOX expression and activity are strictly controlled by environmental (stress) conditions, the AOX2 isoforms of legumes are constitutively expressed (Sweetman et al., 2019a) and can contribute substantially to overall respiration, at least in soybean (Millar et al., 1998).

Recently it was shown that specific AOX1 isoforms of *Arabidopsis thaliana* respond to different sets of organic acids (Selinski et al., 2018), but it is not known whether the same is true of AOX2 isoforms, nor whether there are major differences in activation kinetics between AOX1 and AOX2 subfamilies. Activation kinetics of AOX are very different in sub-mitochondrial particles from different soybean tissues, with cotyledon and root particles exhibiting a tenfold difference in the K_1/2*PYRUVATE*_ (Finnegan et al., 1997). While soybean roots only express AOX2D, cotyledons have high levels of both AOX2A and 2D, but it is unclear whether this explains the different activation kinetics of AOX in two tissues. It is important that we understand the activation kinetics of the different AOX isoforms in legumes, to facilitate the development of more targeted biotechnological approaches for improvement of plant growth and stress tolerance. For example, predicting situations where plant health might be improved *via* AOX engagement, e.g., during abiotic stress (Del-Saz et al., 2016; Vanlerberghe et al., 2016; Watanabe et al., 2016; Dahal and Vanlerberghe, 2018; Sweetman et al., 2019b), or controlling AOX when energy conservation is necessary for improvement of plant productivity (Amthor et al., 2019). Using a system developed specifically for measuring enzyme kinetics of recombinantly expressed AOX proteins (Selinski et al., 2016), we herein describe the differences between pyruvate activation kinetics of soybean AOX1 and AOX2 proteins.

## Materials and Methods

### Generation and Growth of *E*. *coli* Expressing GmAOX Proteins

Mitochondrial target peptide sequences of the *G. max* AOX proteins, GmAOX1, GmAOX2A and GmAOX2D (Whelan et al., 1996) were predicted using Mitoprot (Claros and Vincens, 1996). Primers were designed to amplify the full-length open reading frames, immediately downstream of the mitochondrial transit peptide coding region. Primers also contained restriction sites compatible with pET22b(+): *GmAox1* (F: 5′-CATATGGAGAGCACTTTGGCTTTGTC-3′, R: 5′-GAATTC TTGGCATCATGAGACATAAC-3′), *GmAox2a* (F: 5′-CATATG ATGGTTTCGCCGGCGGA-3′, R: 5′-GTCGACTCATATCCGT GGCAATAAAGACTA-3′), *GmAox2d* (F: 5′-CATATGTCCAC TCTTCCAGAGGTAAA-3′, R: 5′-GAATTCAGCAAAGGCT AGATATCGTT-3′). PCR products were amplified from *G. max* leaf cDNA, inserted into pGEMtEasy *via* A-T ligation (Promega, WI, United States) then digested and cloned into pET22b(+) using standard restriction enzymes (NEB, MA, United States) and T4 ligase (Promega, WI, United States). Resultant constructs: pET22b(+)GmAox1, pET22b(+)GmAox2a and pET22b(+)GmAox2d were confirmed *via* Sanger sequencing (AGRF, SA, Australia), then transformed into *E.coli* strain BHH8 (Selinski et al., 2016) *via* heat-shock. Due to removal of the start codon (as part of the N-terminal target peptide), a new start codon was introduced *via* the *Nde*I restriction site within the F primers. Consequently, the translated peptide sequences contained a methionine that would not typically occur in the mature GmAOX proteins.

### Membrane Vesicle Preparation and Assay Conditions

Methods for bacterial culture, protein synthesis, cell harvesting, and membrane vesicle isolations were carried out as previously described (Selinski et al., 2016). Protein concentrations were determined in a 96-well plate using the Pierce™ BCA Protein Assay Kit (Thermo Fisher Scientific, MA, United States).

Assays were carried out with a Clark type oxygen electrode (Hansatech, Norfolk, United Kingdom), essentially as described by Selinski et al. (2016) but with some modifications. Two electrode chambers were first calibrated and pre-equilibrated with 1 ml NKM buffer (150 mM NaCl, 50 mM potassium phosphate buffer (pH 7.0), 10 mM KCl and 5 mM MgCl_2_). Before beginning the assay, 5 mM DTT, 1 mM KCN and a 10 μl aliquot of inverted membrane vesicles (IMVs; approximately 100 μg) were added, followed by 10 μl of the “effector.” Final pyruvate concentrations ranged between 0.5 and 5,000 μM. After inserting the lids and equilibrating for 3 min, 2.5 mM NADH was used to initiate the reaction, which was inhibited by 1 mM SHAM after 4 min and the residual rate was allowed to proceed for a further 4 min. Each pyruvate concentration was measured in a separate assay; incremental additions were not possible because the activity of membrane vesicles subsided over time. A control reaction containing no pyruvate was run alongside each assay for the same reason, and results were presented as a percentage activation relative to the control. Prior to each set of assays, both chambers were tested with 5 mM pyruvate to ensure the electrodes yielded equivalent rates.

### SDS-PAGE and Immunoblotting

Aliquots of each BHH8 IMV sample were snap-frozen in liquid nitrogen immediately after isolation and stored at −80°C until use for Western blots. Samples were thawed gently, then suspended in loading buffer [62.5 mM Tris (pH 6.8), 30% (v/v) glycerol, 5% (v/v) β-mercaptoethanol, 2% (w/v) SDS, and 0.002% (w/v) bromophenol blue] containing 25 mM DTT. After boiling at 95°C for 2 min and centrifuging briefly, a further 25 mM DTT was added to the supernatant. Samples of 2.5 μg equivalent protein were loaded directly onto 10% SDS-PAGE gels and resolved at 60 V for approximately 60 min, before being transferred to a 0.45 μm nitrocellulose membrane (Trans-Blot Transfer Medium, BioRad, CA, United States). The membrane was incubated with “AOA” antibody raised against *Sauromatum guttatum* AOX in mouse (Elthon et al., 1989) then a peroxidase-conjugated goat anti-mouse IgG (BioRad, CA, United States) and visualized using a chemiluminescent stain (Clarity ECL, BioRad, CA, United States).

### Statistical Analyses

Data were analyzed using SPSS version 25 (IBM). Michaelis Menten constants were determined by non-linear regression. Specific activities of different AOX isoforms were compared using one-way ANOVA with *post hoc* Tukey test.

## Results

**FIGURE 1:**
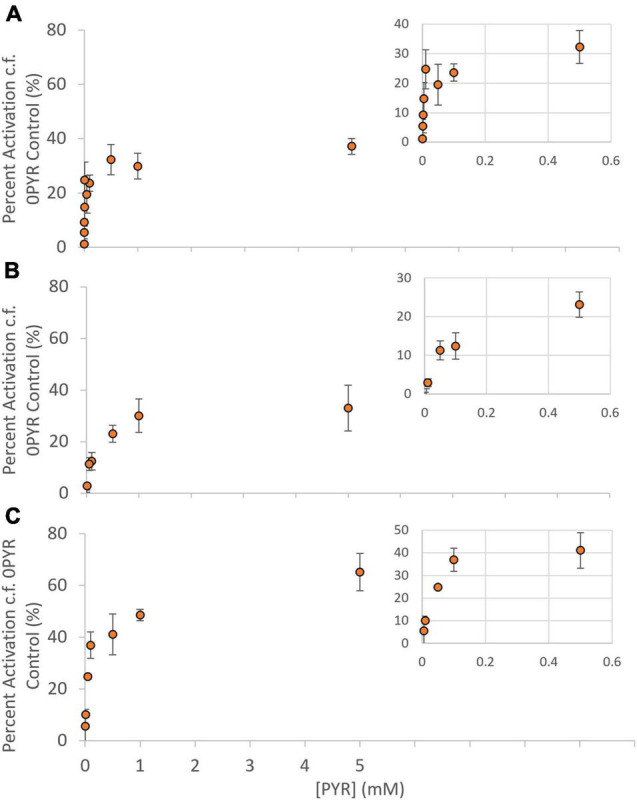
Activation kinetics of AOX1, AOX2A, and AOX2D. Activator saturation curves for **(A)** AOX1, **(B)** AOX2A, and **(C)** AOX2D. Rates presented as Percent Activation; at each pyruvate concentration a simultaneous assay containing no pyruvate (0PYR) was used to calculate activation levels. Each data point represents the mean of measurements taken from three separate IMV preparations (*n* = 3 ± SEM). Michaelis Menten constants were determined by non-linear regression (IBM SPSS v25), and kinetic data summarized in Table 1. Inset: Expanded graph for pyruvate concentrations ≤ 0.5 mM.

*E. coli* BHH8 contains a stable, highly reduced ubiquinone pool due to the lack of cytochrome *bo* quinol oxidase and cytochrome *bd*-1 quinol oxidase, making it ideal for the investigation of heterologously expressed AOX protein kinetics (Selinski et al., 2016, 2017, 2018). Inverted membrane vesicles (IMVs) were prepared from BHH8 cells expressing one of GmAOX1, GmAOX2A or GmAOX2D, and used to measure activation kinetics at different concentrations of pyruvate. Pyruvate activated all three isoforms (Figure 1), but the K_1/2*PYR*_ for GmAOX2A and GmAOX2D (99 and 40 μM, respectively) were much higher than that for GmAOX1 (4 μM) (Table 1). With saturating concentrations of pyruvate, GmAOX1 and GmAOX2A activity was stimulated by about 30%, while GmAOX2D was stimulated by 50%. These maximal activation levels were considerably lower than previous measurements with membrane vesicles containing Arabidopsis AOX isoforms (sixfold increase; Selinski et al., 2016), and with soybean sub-mitochondrial particles (1.85-fold increase in cotyledon SMPs and fourfold increase in root SMPs; Finnegan et al., 1997), suggesting some variability based on expression system and isoform. All of the GmAOX proteins were also activated by 5 mM 2-oxoglutarate (2-OG) (Supplementary Table 1).

**TABLE 1:** Summary of pyruvate activation kinetics for individual soybean AOX isoforms of inverted membrane vesicles and for combinations of AOX isoforms within submitochondrial particles.

	Present study		Finnegan et al. (1997)			
	K_1/2 PYR_ (μM)	Maximum activation (MA,%)	Cotyledon SMPs		Root SMPs	
			K_1/2 PYR_ (μM)	MA (%)	K_1/2 PYR_ (μM)	MA (%)
					
GmAOX1	4	27				
GmAOX2A	99	29	4.5 μM	85%		
GmAOX2D	40	48			51 μM	300%

*Black cells represent absence of GmAOX1 and GmAOX2A protein in root SMPs. Each preparation was repeated three times and mean data used for kinetic calculations.*

**FIGURE 2:**
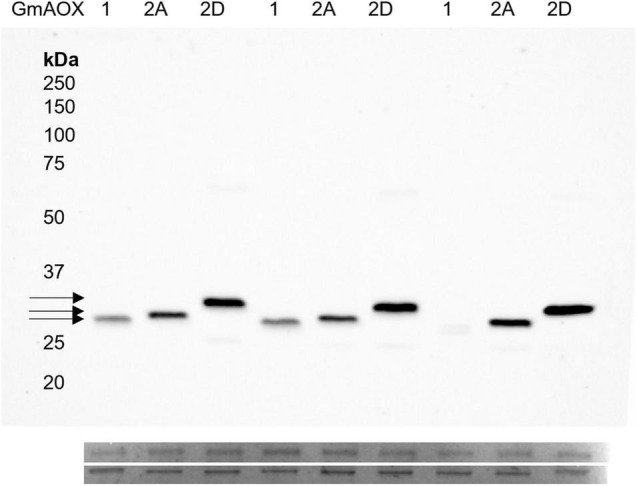
Expression of AOX proteins in BHH8 inverted membrane vesicles. Each lane was loaded with 2.5 μg protein sample, treated with 50 mM DTT. The membrane was probed with AOA antibody, which is non-selective for AOX isoforms (Elthon et al., 1989). Apparent molecular weights were: 28, 29, and 31 kDa for AOX1, AOX2A, and AOX2D, respectively, each lacking the predicted mitochondrial target peptide. Below, two prominent bands from Coomassie stain indicate protein loading.

To ensure that enzyme activities were not influenced by reduction state, DTT was included in all isolation solutions and assay mixtures. Monomeric AOX subunits were confirmed using immunoblots from non-reducing SDS-PAGE (Supplementary Figure 1). GmAOX1, GmAOX2A, and GmAOX2D resolved to different apparent molecular weights: 28, 29, and 31 kDa, respectively (Figure 2), in agreement with apparent Mr measured in soybean mitochondria (Kearns et al., 1992; Finnegan et al., 1997).

**FIGURE 3:**
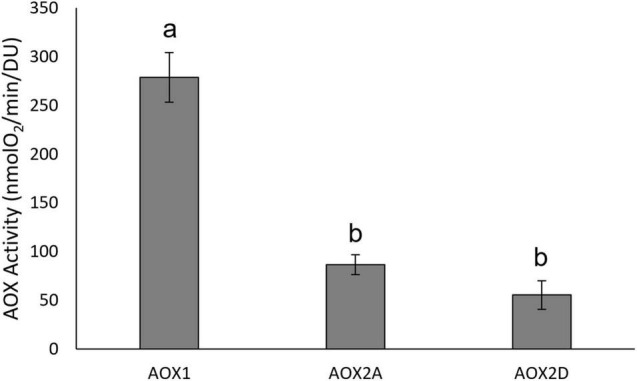
Specific activities of AOX isoforms. Oxygen consumption rates of AOX from inverted membrane vesicles. Rate determined as nmolO_2_/min/μg membrane vesicle protein in the presence of potassium cyanide, DTT and 5 mM pyruvate and initiated with the addition of NADH. Rates were normalized to AOX protein content (density unit reflects the intensity of protein bands in immunoblots, see Selinski et al., 2016) (*n* = 3 ± SEM). Significant differences between isoforms are indicated by different letters, based on one-way ANOVA with *post hoc* Tukey tests (IBM SPSS v25).

The immunoblots showed that GmAOX1 protein was less abundant in membrane vesicle preparations than were the GmAOX2 isoforms (Figure 2). Such differences have been observed previously for Arabidopsis AOX isoforms (Selinski, J. unpublished results). Consequently, specific activities were calculated for the three isoforms based on their relative band intensities on immunoblots of IMV proteins separated by reducing SDS-PAGE (i.e., in the presence of 50 mM DTT; Figure 2). On this basis, it is particularly noteworthy that the specific activity of GmAOX1 (relative level of respiration per unit of AOX protein) was threefold higher than GmAOX2A and fivefold higher than GmAOX2D (Figure 3).

## Discussion

It is always difficult to compare data obtained with a heterologously expressed enzyme to those obtained with native membranes. However, the K_1/2*PYR*_ for GmAOX2D measured here in bacterial membrane vesicles (40 μM) was strikingly similar to that measured previously in sub-mitochondrial particles from soybean root (51 μM; Finnegan et al., 1997), in which only AOX2D is expressed. This gives us some confidence that our results reflect AOX properties *in vivo*. The situation in sub-mitochondrial particles from cotyledons, however, is more complicated since they express multiple AOX isoforms (Finnegan et al., 1997). Interestingly, while the K_1/2*PYR*_ of GmAOX2A (90 μM) was only moderately higher than that of AOX2D (40 μM) in our vesicle system, the K_1/2*PYR*_ for AOX activation in cotyledon sub-mitochondrial particles was much less (4.5 μM; Finnegan et al., 1997), closer to that measured for GmAOX1 in the vesicle system (6 μM). Yet cotyledons of soybean, like young shoots from other legumes, display AOX2A at much higher levels than AOX2D and AOX1 (Finnegan et al., 1997; Sweetman et al., 2019a).

Two possible explanations for this discrepancy come to mind. Firstly, the higher specific activity of GmAOX1 (Figure 3) led it to contribute more strongly to overall AOX activity in cotyledon sub-mitochondrial particles, even though GmAOX2A protein was substantially more abundant, thereby masking the activation kinetics of the GmAOX2 isoforms. However, if this were the case, then a double sigmoid curve of pyruvate activation kinetics would be expected from soybean cotyledon sub-mitochondrial particles, which was not reported by Finnegan et al. (1997). We consider this option as unlikely.

Alternatively, the formation of AOX heterodimers in mitochondria may affect pyruvate activation kinetics. Within the inner mitochondrial membrane, it is generally accepted that active AOX proteins exist as homodimers (Vanlerberghe, 2013). However, this has never been confirmed experimentally and there are hints of heterodimer formation in early work with AOX antibodies (Elthon et al., 1989; Umbach and Siedow, 1993), which have been echoed throughout the literature (Finnegan et al., 1997; Sluse and Jarmuszkiewicz, 1998; Vanlerberghe et al., 2020). It is possible, therefore, that the formation of heterodimers in tissues and cells where multiple isoforms of AOX coexist might affect activation kinetics, with, in the case of soybean, heterodimers being much more sensitive to pyruvate. This would explain the differences observed between the values of K_1/2*PYR*_ values in cotyledon sub-mitochondrial particles and those obtained with AOX proteins expressed singly in the vesicle system. Further work is required to resolve these possibilities.

We also need to consider whether all AOX isoforms reside within the same mitochondrion *in vivo*, since isolated mitochondria originate from multiple cell types when purified from complex tissues such as cotyledons. It is not known whether GmAOX1, GmAOX2A and GmAOX2D co-localize within the same cell types of soybean. However, in *Arabidopsis thaliana* shoots there is indirect evidence for AOX transcript co-localization in some cell types, based on laser microdissection transcriptomics that suggested both At*Aox1a* and *AtAox1b* were present in vasculature cells, while At*Aox1a*, *AtAox1d* and *AtAox2* transcripts were all found in mesophyll (Berkowitz et al., 2021). In addition, interactions between different AOX peptides have been suggested, based on Blue Native (BN)-PAGE experiments with isolated *A. thaliana* leaf mitochondrial proteins; specifically AtAOX1A, AtAOX1B, and AtAOX1D could be found in a complex of approximately 140 kDa (Senkler et al., 2017). The simultaneous expression of multiple AOX isoforms using the experimental system described in this paper could help to confirm, or refute, the formation of heterodimers.

The observation that GmAOX2D was more highly activated by pyruvate than GmAOX2A, at saturating levels of pyruvate, has precedence in the literature. During soybean seedling development, McCabe et al. (1998) observed a shift in transcript and protein level, from predominantly GmAOX2A to predominantly GmAOX2D (AOX1 transcript and protein were low or undetectable), at which point AOX in isolated mitochondria became more highly activated by 2 mM pyruvate. Clearly, the spatiotemporal profiles of specific AOX isoforms can influence regulatory properties of the enzyme in soybean and probably in other plant species. Further research in this area is warranted.

The fact that GmAOX1 has higher specific activity and requires less pyruvate for its activation, compared to its more highly expressed AOX2 counterparts in soybean, may be significant *in planta* because AOX1 is the most stress-inducible isoform in legumes (Sweetman et al., 2019a). GmAOX2D is also stress-responsive and, compared to GmAOX2A, requires about half the amount of pyruvate and is activated to a higher V_*max*_ in the presence of pyruvate. This suggests that stress-induced AOX isoforms respond more quickly to pyruvate (and presumably other organic acid activators) and have a higher inherent capacity than constitutively expressed isoforms such as GmAOX2A that may carry out more ‘house-keeping’ functions in the tissues in which they are expressed.

While it is clear that different AOX isoforms have tissue-specific expression (eg Finnegan et al., 1997), different kinetics of pyruvate activation (this report), and also respond differently to other organic acid activators (Selinski et al., 2018), we still lack precise knowledge of the concentrations of activators *in planta* and how they vary with tissue type and growth conditions. Organic acid concentrations have been estimated in whole tissue extracts from numerous plant species, but for AOX activation it is the intramitochondrial concentrations that are important, and these are very difficult to measure, especially pyruvate given that its import and generation within mitochondria can occur *via* multiple pathways (Le et al., 2021a). The advent of a pyruvate sensor for *in vivo* estimation of pyruvate levels (Arce-Molina et al., 2020) may be of help in this context, although very recent data suggest that even within mitochondria distinct pools of pyruvate exist, and the fate of the different pyruvate pools may be predetermined by source, e.g., import vs *in situ* biosynthesis (Le et al., 2021b).

## Dedication

This article is dedicated to the memory of Jim Siedow, a pioneer of the field of plant respiration, a highly valued colleague, and a very good friend: DD.

## Data Availability Statement

The original contributions presented in the study are included in the article/Supplementary Material, further inquiries can be directed to the corresponding author.

## Author Contributions

DD conceived the project. CS, JS, and TM designed and carried out all experimental procedures. JW contributed to experimental design and provided facilities and guidance. CS drafted the manuscript. JS, JW, and DD edited the manuscript. All authors approved the final manuscript.

## Conflict of Interest

The authors declare that the research was conducted in the absence of any commercial or financial relationships that could be construed as a potential conflict of interest.

## Publisher’s Note

All claims expressed in this article are solely those of the authors and do not necessarily represent those of their affiliated organizations, or those of the publisher, the editors and the reviewers. Any product that may be evaluated in this article, or claim that may be made by its manufacturer, is not guaranteed or endorsed by the publisher.

## Supplementary Material

Supplementary Figure 1 AOX proteins from non-reducing SDS-PAGE.

Supplementary Table 1 Activation of individual soybean AOX isoforms by 5 mM 2-oxoglutarate.
