# Supplementary material for: Legume Alternative Oxidase Isoforms Show Differential Sensitivity to Pyruvate Activation
Source: Front Plant Sci. 2022 Jan 17;12:813691. doi: 10.3389/fpls.2021.813691 (PMC8801435; doi:10.3389/fpls.2021.813691)
Supplement: Supplementary Figure 1 — AOX proteins from non-reducing SDS-PAGE. [file Data_Sheet_1.docx]

Supplementary Material


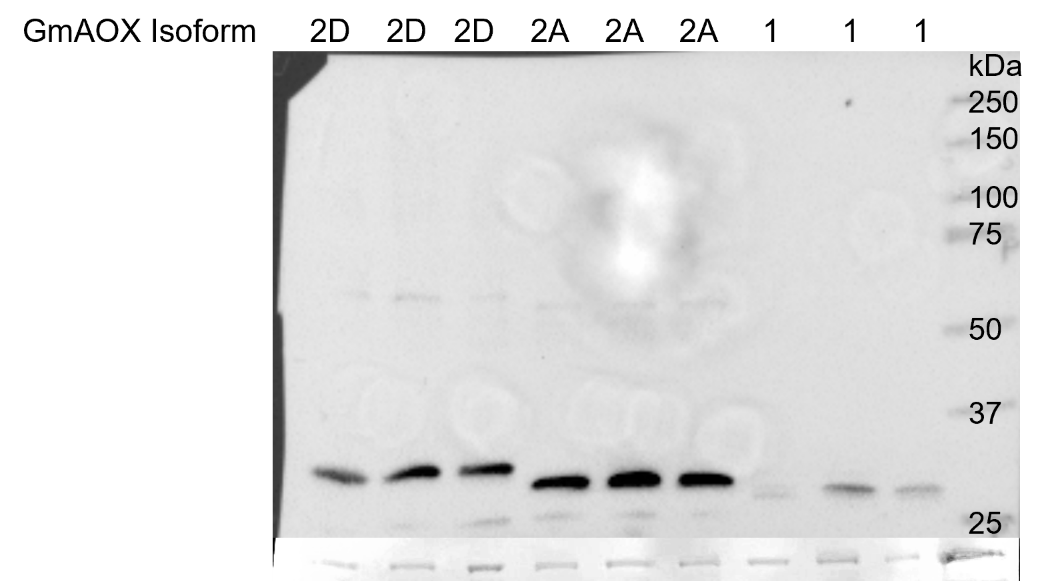


Figure S1: AOX proteins from non-reducing SDS-PAGE. Each lane contained 2.5 ug protein sample in the absence of DTT. The membrane was probed with AOA antibody (Elthon et al. 1989). Apparent molecular weights: 28, 29 and 31 kDa for AOX1, AOX2A and AOX2D, respectively, each lacking the predicted mitochondrial target peptide. Below, a prominent band from Coomassie stain indicates relative protein loading.

**Table S1:** Activation of individual soybean AOX isoforms by 5 mM 2-oxoglutarate. Data were obtained from a single preparation of inverted membrane vesicles, for each isoform.

|  | Activation by 5 mM 2-OG  (%) |
| --- | --- |
| GmAOX1 | 51% |
| GmAOX2A | 27% |
| GmAOX2D | 83% |
